# Effects of Long-Versus Short-Term Exposure to the Mediterranean Diet on Skin Microvascular Function and Quality of Life of Healthy Adults in Greece and the UK

**DOI:** 10.3390/nu11102487

**Published:** 2019-10-16

**Authors:** Markos Klonizakis, Maria G. Grammatikopoulou, Xenophon Theodoridis, Marianne Milner, Yingshan Liu, Michael Chourdakis

**Affiliations:** 1 Department of Nursing and Midwifery, Faculty of Health and Wellbeing, Sheffield Hallam University, Sheffield S10 2BP, UK; mariannebmilner@gmail.com (M.M.); liuyingshan_sylvia@hotmail.com (Y.L.); 2 Department of Nutritional Sciences & Dietetics, International Hellenic University, PO Box 141, Sindos, GR57400 Thessaloniki, Greece; maria@nutr.teithe.gr; 3 Laboratory of Hygiene, Social & Preventive Medicine and Medical Statistics, Department of Medicine, Faculty of Health Sciences, Aristotle University of Thessaloniki, University Campus, GR54124 Thessaloniki, Greece; xenofontheodoridis@gmail.com (X.T.); mhourd@gapps.auth.gr (M.C.); 4 Department of Medicine, Faculty of Health Sciences, University of Thessaly, Larissa, Greece

**Keywords:** Mediterranean diet, microcirculation, quality of life, cardiovascular disease, dietary intervention, nutrition therapy, clinical trial

## Abstract

The beneficial effects of the Mediterranean diet (MD) adherence in reducing cardiovascular disease (CVD) risk and improving CVD-related physiological indices have been well-documented. However, the exact MD adherence duration needed for these effects to occur is under-researched. The aim of the present, two-arm, two-site study clinical trial was to assess the effects of long- vs. short-term MD adherence on the skin microvascular circulation, and quality of life. Two groups were recruited, one being long-term MD adherers (>5 years; from Greece; control group), and one of the non-adherers (from the UK), with the latter participating in a four-week MD intervention (intervention group). Our main outcome was skin microvascular function assessed by cutaneous vascular conductance (CVC). Secondary outcomes included quality of life, dietary intake, blood pressure and lipidemic profile. At the end of the intervention, both groups had high MD adherence. For the intervention group, significantly improved post-intervention CVC values were noted concerning the initial peak phase (2.0 ± 0.6 vs. 2.8 ± 0.8; *p* < 0.05). CVC values of the control group, were however higher at the plateau phase in comparison to the intervention group (intervention end; 3.8 ± 0.8 vs. 3.1 ± 1.2; *p* < 0.05). As per QoL, the physical domain was improved post-intervention (13.7 ± 1.2 vs. 15.9 ± 1.2; *p* < 0.05). No differences were observed in the lipidemic profile between groups, or between the baseline and final intervention phases. The findings indicate that although short-term MD adherence is effective in improving certain microvascular physiological properties and QoL domains, there is room for additional improvement, observed in long-term adherers. Our findings are important in the design of future, MD-based, lifestyle interventions, with the advisable durations differing between target groups.

## 1. Introduction

Cardiovascular disease (CVD) is the biggest preventable cause of mortality in the western world [1]. Interestingly, despite treatment innovations and efficacy, the number of people who are either at risk, or suffering from any CVD form is following an increasing trend, year-by-year [2]. Therefore, the importance of reducing CVD and CVD risk remains high. Lifestyle interventions either based on diet [3], exercise [4], or in combination [5], have been proposed as a means for improving cardiovascular health in clinical and healthy-but-at-risk populations. Amongst those, the ones that markedly stand out are based on the Mediterranean diet (MD) model [5,6,7,8]. The MD is a collective term for the dietary patterns consumed by the people inhabiting the Mediterranean countries. It involves increased consumption of olive oil, vegetables, legumes, oily fish, cheese, nuts and moderate amounts of white meat and wine [9,10]. MD appears to provide clinical benefits in reducing the risk for CVD, diabetes and cancer [7]. Although the MD benefits are considered to be significant, there are certain aspects that need further clarification—for example, it is not known as of whether the improved physiological effects in skin microvascular health from the long-term adherence to the MD are greater to those observed following a short-term adherence (as someone would expect them to be) and if they are, to what extent. This is important, as it is now known that changes in the skin endothelial function, mirror if not precede those observed in large arteries [11]. Consequently, knowing the effects of an MD-based dietary intervention on skin microcirculation, may well influence clinicians’ recommendations and public health policies involving MD as a "healthy diet" option, because it will determine the duration of periods of full implementation and maintenance that should be targeted for. It will also help in determining whether there are limitations to the benefits offered by the MD alone, and if additional lifestyle arms (e.g., exercise, sedentary behavior reduction, etc.) would be necessary to achieve a greater CVD risk-reduction target. In this context, the present study was designed aiming to define the effects of short-term MD adherence on CVD risk using as a proxy endothelial dysfunction, compared to long-term MD adherence, among healthy, sedentary, adult populations. We hypothesized that people adhering to the MD for longer would have a better endothelial function, defined by NO-bioavailability and axon reflex indicators (see *Methods and Materials* section). Additional outcomes included changes in the quality of life (QoL), anthropometry and blood lipid profile. For the purpose of the study, we recruited one Mediterranean (Greek) population that has followed the MD for a long period of time (>5 years) and for which MD is part of their culinary traditions (control group), and one population residing far from the Mediterranean sea (British), that has followed the MD for a short time period (4 weeks), without being previously accustomed to it (intervention group). 

## 2. Methods and Materials

### 2.1. Study Design and Ethics

This was a two-centre (Sheffield, UK and Thessaloniki, Greece), parallel-arm, open-label intervention trial. The protocol was approved by the Sheffield Hallam University Ethics Review Committee (HWB-2016-17-S&E-44) and the Aristotle University of Thessaloniki Research Ethics Committee (251/26.2.18). The protocol was registered at the Thai Clinical Trials Registry (TCTR20190305002), via the World Health Organization trials registry platform. All experiments complied with the current laws of the countries in which they were performed, and all participants provided written consent to participate in the study.

### 2.2. Study Population and Procedures

For the study, two population groups were recruited, one consisting of long-term MD adherers (control group), recruited in Greece, and one of the non-adherers (intervention group), recruited in the UK, scheduled for participation in an MD intervention. Both groups (18–75 years old) consisted of healthy, sedentary adults (controls: Thessaloniki, Greece, *n* = 40, and intervention: Sheffield, UK, *n* = 34) (Table 1). Participants were recruited through local community groups, social and mass media and word of mouth. Posters were placed around regional hospitals and university campuses. 

Inclusion criteria were: (1) Being an adult; (2) abstaining from any medication that could influence cardiovascular function; (3) being willing to participate; (4) adhering to the MD for over five years (self-reported; control group); and (5) MD non-adherence (intervention group). The 5-year cut-off for the control group was used, as it was considered that it would be sufficientfor the long-term MD benefits to be established.

Exclusion criteria were: (1) Participation in regular physical exercise (>60 min/week of structured or planned physical activity; known to affect positive microvascular integrity); (2) diagnosis of any chronic disease that could affect vascular function (e.g., diabetes, CVD, and hypertension); (3) pregnancy; and (4) smoking. No financial incentives were given to any of the participants to increase participation. 

**Figure 1:**
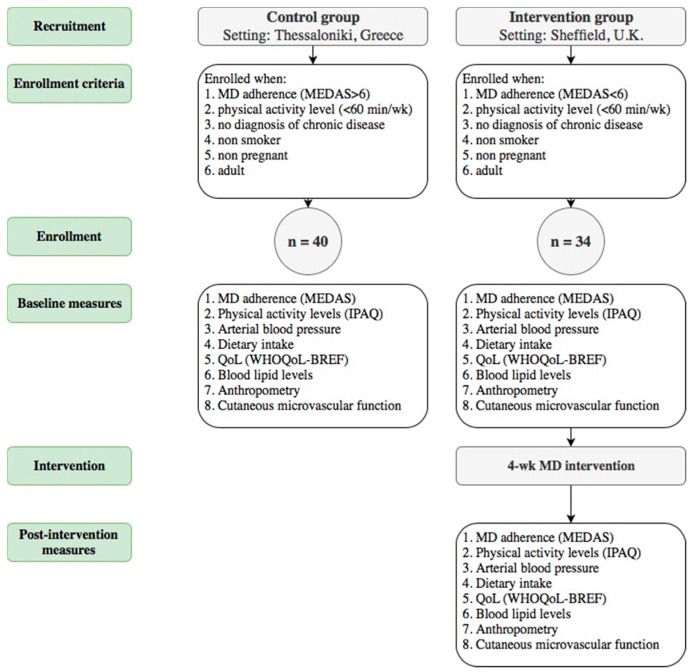
Flowchart of the selection of participants and the study procedure.

A flowchart of the recruitment, screening and study procedures performed in each group is presented in Figure 1. Following the provision of written consent, controls were invited to the Medical School, Faculty of Health Sciences of the Aristotle University of Thessaloniki for a single, 70-min session. In parallel, screening for participation in the intervention group took place at the Centre for Sport and Exercise Science of Sheffield Hallam University for an identical set of baseline assessments. The intervention group then followed the MD for four weeks, after which, all measurements were repeated. Participants in both groups were asked to refrain from drinking caffeinated drinks for a period of three hours prior to the assessments.

#### 2.2.1. Mediterranean Diet Adherence 

MD adherence was assessed with the MEDAS questionnaire, a validated MD adherence screener [12] that has been previously used on the Greek population [13]. The MEDAS assesses the frequency of intake of specific food-groups associated with the MD, including olive oil, wine, vegetables and fruits, legumes, fish and shellfish against the frequency of intake of food groups associated with a more westernized diet regime, like sweets and candies, animal fat, carbonated beverages, red meat, etc. A score greater than 6 indicates increased MD adherence, and subsequently, a score of less than 6 reflects MD non-adherence. Subsequently, a MEDAS score >6 was a selection criterion for the Greek cohort and a MEDAS <6 for the UK sample. This way, the Greek sample consisted of long-term MD adherers and the UK cohort of non-adherers who would participate in the short-term MD intervention protocol. For a more thorough dietary assessment, each participant also completed a 48h dietary recall questionnaire [14] for each study visit, with controls completing one dietary recall and participants in the intervention group completing one at baseline and one post-intervention. Collected data were entered into a dietary analysis software, which incorporated the “McCance and Widdowson’s UK Composition of Food Database” [15] within its databank (Nutritics Ltd., Version 1.7, Dublin, Ireland). 

#### 2.2.2. Physical Activity Levels, Anthropometry and Quality of Life 

Physical activity levels were assessed as metabolic equivalents of task minutes via the International Physical Activity Questionnaire-SF (IPAQ) [16], to ensure that all participants met the sedentary requirements set for the study. Measured anthropometric indices included height, weight, calf and waist circumference measured, as well as the calculation of body mass index (as body weight (kg)/height (m)^2^. QoL was assessed via the WHOQoL-BREF [17] questionnaire in the Greek (control group) and English language (intervention group) [16,18].

#### 2.2.3. Blood Lipid Profile and Arterial Blood Pressure 

Participants’ gender was recorded, and heart rate (HR), systolic (SBP) and diastolic blood pressure (DBP) were measured after 15 minutes of rest, three times, at one-minute intervals, with the mean value being used (left arm; Dinamap Dash 2500, GE Healthcare, USA). Finger-prick blood-capillary samples were also obtained using a Cholestec LDX system (Alere Cholestech LDX® system; Alere Inc., San Diego, CA, USA) to determine values for Total Cholesterol (TC), High Density Lipoprotein (HDL) and TC/HDL, as previously described [8].

#### 2.2.4. Physiological Measures of CVD Risk—Laser Doppler Flowmetry (LDF) 

For the purposes of this study, we concentrated on endothelial dysfunction, as it is a common phenomenon of all major CVD risk factors (e.g., high blood cholesterol, hypertension, diabetes, etc.). To determine cutaneous microvascular function, we used LDF, combined with local heating of the skin, as previously described in the literature [3]. This allows us to obtain information primarily on neurone-mediated axon reflex function (initial peak; [19]) and NO bioavailability (secondary rise and plateau phase; [19]). Tests in both groups were conducted in temperature-controlled rooms, with an ambient temperature of 22–24 °C, as previously described by our lab [3]. Tests in all participants were undertaken in the upper right forearm in skin sites without visible veins, body hair, and damaged or irritated skin. The testing site was sterilized, and then the LDF probe was attached to the skin. Local thermal hyperemia was induced using a heating disk (Model 455, Perimed AB) connected to a heating unit (Model 5020, Perimed AB). LDF signals were recorded using PeriSoft for Windows 9.0 software (PeriSoft for Windows 2.50; Perimed AB). During each test, the resting HR and blood pressure were recorded from the left arm at regular intervals using a patient monitoring device (Dinamap Dash 2500, GE Healthcare, Florence, SC, USA). The LDF assessment protocol began with the recording of baseline skin blood flow data for a 5 min period with the local heating disk temperature being set at 32 °C. Then, in order to induce rapid local heating, the temperature of the probe was increased by 1 °C every 10 s until the local heating disk reached 42 °C, which was maintained for a period of 25 min. Then the test was concluded, with the temperature being rapidly increased to 44 °C for the final 10 min, to support the assessment of maximal vasodilation. Measurement phases were then defined (Table 2). The LDF values divided by the corresponding mean arterial pressure to give the cutaneous vascular conductance (CVC) in arbitrary perfusion units (APU)/mm Hg. The mean arterial pressure was calculated from SBP and DBP. The data presented as raw CVC and CVC was normalized to maximum (%CVC_max_ = ((CVC/CVC_max_) × 100)). The resting HR and BP of participants were calculated from the average of measured HR and BP throughout the LDF recording period.

#### 2.2.5. Mediterranean Diet Intervention 

Intervention group participants were given a food “starter” pack that included amongst others brown rice, brown pasta, lentils, olive oil, nuts, chopped tomatoes and tomato puree. They were also given an information booklet, guidelines on which foods constitute the MD, and a recipe booklet to support the transition of their current diet to the MD prototype. The research team also created and moderated an “invitation-only” social media group, to support intervention group participants, in addition to the provision of extensive support via regular phone-calls and e-mail exchange. To achieve a low attrition rate, we used our ‘six pillars of adherence’ framework, based upon ‘social support’, ‘education’, ‘reachability’, ‘small groups intervention implementation’, ‘reminders’, and ‘simplicity’, which has been previously applied with excellent results in lifestyle interventions (achieving more than 90% of retention and 79% of completion rates) i.e., [3,4].

### 2.3. Statistical Analysis

For our sample size calculations we used commercial software (G*Power 3.1.7, HHU of Düsseldorf), using initial peak CVC as the primary outcome and data from our previous studies [3]: For an estimated difference of 0.6 AU/mmHg between groups and an estimated standard deviation of 0.9 AU/mmHg, we calculated that we would need 34 people per group. Over-recruitment was implemented in the control group, to account for any last-minute cancellations and avoid missing recruitment targets within our designated timeframe. 

Independent t-tests were performed on physical characteristics (stature, body mass, BMI, systolic and diastolic blood pressure), nutritional data (energy Intake, carbohydrates, protein, total fat, saturated fat, MUFA, PUFA, olive oil, fruit, vegetables, and fiber), raw CVC and %CVC_max_ (baseline, initial peak, and plateau), WHOQoL, IPAQ sitting time, MEDAS score and lipidemic profile (TC, HDL, and TC/HDL) data between groups. Dependent t-tests were performed to determine pre-to-post changes for the intervention group. To satisfy normality, logarithmic transformations were undertaken for several variables (carbohydrate and saturated fat intake, BMI, TC/HDL, baseline %CVC_max_ and initial peak %CVC_max_). Where homogeneity of variance or normality could not be assumed (i.e., in raw CVC initial peak and plateau (pre-intervention), and initial peak %CVC_max_), the Mann–Whitney U test was undertaken to assess between-groups comparisons; the Wilcoxon signed-rank test was performed for within-group analyses.

Statistical analysis was performed using SPSS (IBM SPSS Statistics Version 22 (IBM United Kingdom Limited, Hampshire, UK). Statistical significance for the test was set at *p* ≤ 0.05. Values are presented as mean ± standard deviation (SD), unless otherwise stated.

## 3. Results

### 3.1. Anthropometric and Physical Activity Measures

No differences were observed in the BMI, height, weight, fat mass, resting HR, SBP, calf and waist circumferences, waist/hip ratio, or IPAQ measures either between groups, or for the intervention group, pre- and post-intervention (Table 1). However, a different gender ratio was recruited in the two groups.

### 3.2. MD Adherence and Dietary Components

The dietary intake of each group is summarized in Table 3. Mean MEDAS score increased significantly in the intervention group (*p* < 0.05) at the intervention end. Moreover, a between-groups comparison suggests that at the intervention end, the intervention group was more compliant to the MD than the control group (*p* < 0.05; Table 3). At the intervention end, controls consumed more MUFAs (*p* < 0.05), total fat (*p* < 0.05), saturated fat (*p* < 0.05) and olive oil (*p* < 0.05). They also consumed fewer carbohydrates, without reaching statistical significance. On the other hand, the intervention group consumed more fiber and proteins (*p* < 0.05; Table 3).

### 3.3. Cutaneous Vascular Conductance

Baseline values remained unchanged between visits for the intervention group. No statistically-significant differences were observed between the control and intervention groups (intervention end). 

For initial peak, raw CVC values were significantly improved at the end of the 4-weeks, for the intervention group (*p <* 0.05; Table 4), while no statistically-significant differences were observed following a between-groups comparison. However, the between-groups comparison revealed a statistically-significant difference for %CVC_max_, with the intervention group reaching a higher percentage of their maximum CVC value at that point (*p* < 0.05; Table 4).

For the plateau stage, the raw CVC of the control group was significantly higher to that of the intervention group (intervention end values; *p* < 0.001; Table 4). No statistically-significant differences were observed for %CVC_max_, either between-groups or for the intervention group before and after the intervention.

### 3.4. Quality of Life and Lipidemic Profile

No differences were noted between groups, with the exception of the social domain (*p* < 0.05). An improvement was also observed at the end of the intervention among intervention group participants, with regards to the physical domain (*p* < 0.05) (Table 5). No differences were observed either between-groups, or in the intervention group between the two study visits concerning the lipidemic profile of the sample (Table 5).

## 4. Discussion

The present study is the first to compare endothelial microvascular physiology and QoL between long- and short-term exposure to the MD. The results showed that short-term MD adherence is effective in ameliorating selected markers of endothelial function and QoL domains among MD-naïve participants. Although the MD-imposed microcirculatory benefits are well-described for medium [5] and short-term [3] compliance of the MD, we are yet to establish if longer-term consumption can bring additional microvascular physiological benefits. This knowledge would be important to tailor therapies for certain diseases or conditions; for example, a reduction in the production of nitric oxide (NO) has been associated with higher CVD risk [20], while it is known that ageing has an effect on vascular tone, maintaining the axon-mediated vascular vasodilation and NO production [21]. Therefore, if for instance short-term MD adherence can induce benefits on NO bioavailability, but not on the vascular tone, then this would probably mean that longer-term interventions would be more appropriate for older or other clinical populations, for which the vascular physiology is similarly affected (e.g., systemic sclerosis) [22].

### 4.1. Quality of Life

Our findings suggest that the study participants in both groups maintain a happy and healthy life with improvements being noticed in the intervention group at the end of the intervention. Participants in the control group in particular, tend to score better in the WHOQoL compared to the average values of the Greek population reported by other studies, at the majority of domains (i.e., physical domain: 16.2 (1.8) vs 15.2 (2.2); [21]), while the UK cohort matching those at the end of the intervention [21]. Although the overall quality of life is affected by an array of parameters (including demographic, developmental, psychological, and biological ones) [23], it can be argued that any observed changes in a particular domain can be attributed to a specific parameter that changed, if the others remain unaltered. Our study confirms the general notion derived from MD-based work [7] and studies based on other “healthy” diets [24] that a “healthy” dietary regime brings higher scores for health-related QoL, with particular benefits on physical and emotional well-being [24].

This is the first study, undertaken in a community-setting, to suggest that QoL benefits are prompt to appear, only four weeks after the adoption of the MD. This is an important finding, considering that the adaptation to a new dietary regime is not an easy process, and there are many challenges (financial, habitual and societal) to be faced by all parties involved [25], despite the fact that in most cases those adapting do recognize the benefits that come with it [26]. Our findings, therefore, can be used as a supporting element for both dieticians/nutritionists who make the suggestion for the dietary change to the MD and those at the receiving end, but also for those involved in public health policy making. 

### 4.2. Compliance to the MD

Our study verified that the MD diet could be adopted and adhered to by MD-naive, non-Mediterranean populations [27]. Our results suggest that the intervention group participants managed to adapt themselves to the new diet quite quickly and successfully between the two visits (Table 2). Nevertheless, it appears the type of MD that they consumed was different to the one followed by our long-term group participants, being abundant in proteins and fiber, with less fat, despite the statistically-significant increase noted in the olive oil consumption (Table 3). On the other hand, the control group consumed more fat, primarily due to the greater intake of virgin olive oil, which affects the MUFA intake [28]. These differences under the MD dietary pattern, cannot be considered as a surprise: It is well accepted that such variations do exist, and that MD is a collective term of multiple dietary protocols with similar ingredients at varying amounts [10,29]. Although the comparison between populations consuming the MD and the use of different methods of measuring adherence is a highly-debated topic [25,30], we can be confident that both groups followed a similar dietary pattern, albeit with some differences in the fat sub-types’ intake. Furthermore, it is accepted that habitual MD consumption in the Mediterranean region is usually associated with greater PUFA and MUFA intake [31] and therefore, such a finding should have been expected by a Greek population, like the controls group herein.

According to the latest representations, the MD food pyramid also includes regular physical activity and adequate rest, whereas, in our study, only sedentary participants were considered eligible, in an effort to control for this factor [32,33]. Thus, it should be expected that in a more “pragmatic” clinical trial, including more appropriate physical activity patterns, the effects of MD adherence would be further improved in several endpoints, including the QoL [34].

### 4.3. Microvascular Function 

This is the first study to suggest that although a short-term lifestyle intervention based on the MD can bring quick benefits in regards to the axon-reflex microcirculatory function (as is described by an improvement in “initial peak” stage), an improvement in NO bioavailability (as this is described by “plateau” stage) is not also observed, and remains statistically-lower to the values observed in the control (long-term MD adherers) group. It can be argued that the statistically-significant observed differences, may not be solely due to the difference in the duration of the consumption of the MD between the two groups, but also due to the different constitution of the MD. Indeed, recent evidence suggests that that the high intake of MUFA (which was the case for our longer-term population) comes with greater cardioprotective properties [28] and can lower CVD risk especially in high-risk populations [35]. This would on this own, probably reflect positively at microcirculatory function. Nevertheless, our control group did have a high saturated fat intake -markedly larger to the one received by the short-term population- which in turn is known to increase CVD risk [31,35] and affect circulatory function at all levels. In that case, considering that: (a) Plant- and sea-derived PUFA also have cardioprotective properties [31,36]; and (b) it is quite improbable that the protective benefits of MUFA can cancel out the saturated fats’ negative ones completely, it is tempting to postulate that the differences between the two groups can primarily be attributed to the difference between the duration of the consumption of the MD. Unfortunately, a further breakdown of the participants’ consumption patterns to quantify green vegetable intake was not accounted for when designing the protocol herein. Thus, we ought to consider that increased green vegetable intake might also induce improvements in the NO profile of the Greek cohort, as suggested [37,38]. Further interventional studies, in which groups will be followed for longer-term periods will be able to confirm this postulation. 

Our findings may have an implication in the way and the duration that MD-based lifestyle interventions are prescribed in clinical and pre-clinical groups. For example, in T2DM [39] and systemic sclerosis [22,40], microcirculatory changes are more prominent in the axon-reflex. Therefore, it makes sense that for these populations, we have at least a short-term MD intervention (ideally in combination with other lifestyle elements, such as exercise and smoking cessation) to be advised to introduce quicker physiological benefits. The advice, however, would be different in older populations (clinical or not), since it is well-accepted that ageing has a two-fold, negative effect on both microcirculatory NO production and axon-reflex vasodilation. For those populations, a longer-term strategy would be more appropriate, having the added-benefit of the MD-associated, systemic physiological benefits [39].

### 4.4. Experimental Considerations 

Despite its importance, our study also has a number of limitations. Due to operational difficulties, it was not possible to have an equal ratio of genders in the two study groups. This might have had an effect on the CVD risk between the two groups given the different phenotypes associated with distinct ethnic backgrounds [41]. However, both groups have a very good match regarding most of their main characteristics. Additionally, it was not possible to have baseline assessments for the control group. This would have required a larger amount of resources to the ones that our study had. Further studies may address this limitation. We also used 2-d diet diaries instead of the gold standard 7-d weight food diary. We opted for that choice to reduce participants’ burden and reduce likely dropouts/complete recruitment within our limited time period. Moreover, this remains a validated collection method of dietary data.

Additionally, it should be mentioned that some of the exerted beneficial health effects might also be due to the overall Mediterranean lifestyle, including adequate sun exposure and increased vitamin D synthesis, and not to the diet per se [42,43]. This is an important point to consider when accounting for environmental factors that might affect the results in the intervention arm. Finally, although our sample was recruited through advertising, it may not be representative of either the Greek or the British population. Nevertheless, our findings can be considered as indicative, paving the way for a larger, longer-term trial.

## 5. Conclusions

Our work is the first to compare the skin microvascular and QoL effects between long- and short-term exposures to the MD. Our findings suggest that although MD brings quick benefits concerning axon-reflex mediated vasodilation (which might be sufficient for certain clinical populations, such as T2DM and systemic sclerosis), longer interventions are required for the full range of microcirculatory physiological benefits to be experienced. At the same time, however, QoL benefits are experienced within a relatively short period (four weeks), which may prove decisive for all those attempting (and struggling) to accustom themselves in a new, culturally different dietary regime. Additionally, the present study paves the way for further research of longer duration, in both clinical and healthy populations.

## Figures and Tables

**Table 1:** Baseline characteristics of participants.

	Control Group (*n* = 40)	Intervention Group (*n* = 34)	
	Single visit	Baseline	4 Weeks
Gender (Male/Female ratio) (*n*)	29/11	11/23 *	
Age (years)	37 ± 11	40 ± 17	
Height (cm)	171 ± 9	169 ± 1	
IPAQ Sitting Time (min)	480 ± 270	475 ± 120	465 ± 118
Body Weight (kg)	68.9 ± 15.1	72.0 ± 12.9	71.0 ± 11.3
BMI (kg/m^2^)	23.4 ± 4.6	25.2 ± 6.1	24.6 ± 5.5
Waist circumference (cm)	78 ± 15	81 ± 9	80 ± 8
Waist/hip ratio	0.65 ± 0.1	0.66 ± 0.1	0.64 ± 0.2
Physical activity occupational category (sedentary/low/moderate/intense) (*n*)	28/8/4/0	24/7/1/0	
Calf circumference (cm)	49 ± 6	51 ± 6	50 ± 5
Resting heart rate (bpm)	70 ± 10	69 ± 8	68 ± 7
Resting SBP (mm Hg)	120 ± 11	118 ± 12	117 ± 11
Resting DBP (mm Hg)	78 ± 8	75 ± 11	74 ± 10
Body Fat (% of body weight)	30.0 ± 9.0	29.0 ± 9.0	28.1 ± 8.2

BMI: Body mass index; bpm: Beats per minute; DBP: Diastolic blood pressure; IPAQ: International Physical Activity Questionnaire; IQR: Interquartile range; SBP: Systolic blood pressure; SD: Standard Deviation. Data are presented as n, mean ± SD, or IQR (range). * Significantly different between groups (*p* < 0.05).

**Table 2:** Laser Doppler flowmetry measurement phase definition.

Measurement Phase:	Time Point:
Baseline	The arithmetical mean of the last 2 min of the first 5 min period.
Initial peak	The arithmetical mean of the highest consecutive 30-s period within the distinct initial hyperemic response.
Plateau	The arithmetical mean of the last 2 min of heating at 42 °C.
Maximum	The arithmetical mean of the last 2 min of heating at 44 °C.

**Table 3:** Dietary intake and compliance with the Mediterranean diet between groups and time-points (mean ± SD).

MD Adherence, Food-Group and Nutrient Intake	Control Group (*n* = 40)	Intervention Group (*n* = 34)	
	Single Visit	Baseline	4 Weeks
MEDAS score	9 ± 1	5 ± 1	11 ± 1 *^,++^
Energy Intake (kcal/day)	1742 ± 353	1865 ± 323	1732 ± 432
Carbohydrates (g/day)	155 ± 45.6	213 ± 33	172.3 ± 55 *
Fiber (g/day)	17.9 ± 8.4	17.0 ± 6.1	23.5 ± 7.2 *^,++^
Protein (g/day)	56.7 ± 18.7	70.5 ± 20.5	74.6 ± 20.1 ^++^
Fat (g/day)	102.8 ± 21.5	86 ± 21	71 ± 23 *^,++^
Saturated fat (g/day)	24.2 ± 8.2	32.0 ± 7.5	20 ± 9.1 *^,+^
MUFA (g/day)	62.4 ± 11.0	27.2 ± 9.0	32.3 ± 9.5 ^+^
PUFA (g/day)	12.0 ± 4.3	11.9 ± 4.4	13.0 ± 6.5
Olive oil (servings/day)	4.5 ± 0.5	0.4 ± 0.4	2.1 ± 1.5 *^,++^
Fruit and vegetables (servings/day)	7.2 ± 2.1	3.3 ± 2	6.9 ± 3.3 *

* *p* < 0.05 within groups; ^++^ *p* < 0.01 and ^+^ *p* < 0.05 between groups. Data presented as mean ± SD. For olive oil, serving is calculated as tablespoon, for fruits and vegetables as 80 g. Between groups comparison assesses differences between the control group, and the intervention group at the end of the trial. MD: Mediterranean diet; MEDAS: Mediterranean diet adherence screener; MUFA: Mono-unsaturated fatty acids; PUFA: Poly-unsaturated fatty-acids; SD: Standard deviation.

**Table 4:** Microcirculatory results between long- and short-term Mediterranean diet adherers (mean ± SD).

Measure	Control Group (*n* = 40)	Intervention Group (*n* = 34)	
	Single Visit	Baseline	4 Weeks
Baseline CVC (APU/mmHg)	0.1 ± 0.1	0.1 ± 0.1	0.1 ± 0.2
Initial peak CVC (APU/mmHg)	2.7 ± 0.6	2.0 ± 0.6	2.8 ± 0.8 ^+^
Plateau CVC (APU/mmHg)	3.8 ± 0.8	2.2 ± 0.6	3.1 ± 1.2 *
Baseline CVC_max_ (%)	3.0 ± 2.3	8.9 ± 5.2	7.3 ± 8.5 *
Initial peak CVC_max_ (%)	56.7 ± 8.2	73.1 ± 15.2	75.5 ± 16.1 *
Plateau CVC_max_ (%)	79.4 ± 9.6	86.1 ± 16.2	82.8 ± 9.7

APU: Arbitrary perfusion units; CVC: Cutaneous vascular conductance; SD: Standard deviation. * *p* < 0.001 for the between groups’ comparison. + *p* < 0.05 for the intervention group comparison between time-intervals.

**Table 5:** Quality of life and Lipidemic profile of participants in both groups (mean ± SD).

Variables	Control Group (*n* = 40)	Intervention Group (*n* = 34)	
	Baseline	Baseline	4 weeks
***QoL domains***			
Physical	16.2 ± 1.8	13.7 ± 1.4	15.9 ± 1.2 ^+^
Psychological	15.5 ± 2.3	13.9 ± 1.8	14.4 ± 2.3
Social	15.6 ± 2.2 *	13.1 ± 3.9	14.6 ± 1.7
Environmental	13.9 ± 1.9	13.3 ± 1.6	13.7 ± 1.6
***Lipidemic profile***			
TC (*mmol/L*)	4.1 ± 1.1	4.1 ± 0.6	4.2 ± 0.6
HDL (*mmol/L*)	1.6 ± 0.7	1.4 ± 0.5	1.5 ± 0.3
TC/HDL (*ratio*)	2.8 ± 1.1	3.6 ± 1.9	3.0 ± 0.9

* *p* < 0.05 for between groups comparison; ^‡^ *p* < 0.05 within group comparison. Data presented as mean ± SD. Between groups comparison assessed differences between the control and intervention group at the end of the trial. HDL: High-density cholesterol; MD: Mediterranean Diet; QoL: Quality of Life; SD: Standard Deviation; TC: Total cholesterol.
